# The Influence of Gestational Diabetes on Neurodevelopment of Children in the First Two Years of Life: A Prospective Study

**DOI:** 10.1371/journal.pone.0162113

**Published:** 2016-09-07

**Authors:** Shirong Cai, Anqi Qiu, Birit F. P. Broekman, Eric Qinlong Wong, Peter D. Gluckman, Keith M. Godfrey, Seang Mei Saw, Shu-E Soh, Kenneth Kwek, Yap-Seng Chong, Michael J. Meaney, Michael S. Kramer, Anne Rifkin-Graboi, GUSTO study group

**Affiliations:** 1 Department of Obstetrics & Gynaecology, Yong Loo Lin School of Medicine, National University of Singapore, National University Health System, Singapore, Singapore; 2 Singapore Institute for Clinical Sciences, Agency for Science and Technology Research (A*STAR), Singapore, Singapore; 3 Department of Biomedical Engineering and Clinical Imaging Research Centre, National University of Singapore, Singapore, Singapore; 4 Department of Psychological Medicine, Yong Loo Lin School of Medicine, National University of Singapore, National University Health System, Singapore, Singapore; 5 Liggins Institute, University of Auckland, Auckland, New Zealand; 6 MRC Lifecourse Epidemiology Unit and NIHR Southampton Biomedical Research Centre, University of Southampton and University Hospital Southampton NHS Foundation Trust, Southampton, United Kingdom; 7 Saw Swee Hock School of Public Health, National University of Singapore, Singapore, Singapore; 8 Department of Pediatrics, Yong Loo Lin School of Medicine, National University of Singapore, National University Health System, Singapore, Singapore; 9 Department of Maternal and Fetal Medicine, KK Women’s and Children’s Hospital, Singapore, Singapore; 10 Department of Psychiatry, Faculty of Medicine, McGill University, Montreal, Canada; 11 Department of Epidemiology, Biostatistics and Occupational Health, McGill University Faculty of Medicine, Montréal, Canada; 12 Department of Pediatrics, McGill University Faculty of Medicine, Montréal, Canada; Iran University of Medical Sciences, ISLAMIC REPUBLIC OF IRAN

## Abstract

**Objective:**

Analyze the relation of gestational diabetes and maternal blood glucose levels to early cognitive functions in the first two years of life.

**Methods:**

In a prospective Singaporean birth cohort study, pregnant women were screened for gestational diabetes at 26–28 weeks gestation using a 75-g oral glucose tolerance test. Four hundred and seventy three children (n = 74 and n = 399 born to mothers with and without gestational diabetes respectively) underwent neurocognitive assessments at 6, 18, and/or 24 month, including electrophysiology during an attentional task and behavioral measures of attention, memory and cognition.

**Results:**

Gestational diabetes is related to left hemisphere EPmax amplitude differences (oddball versus standard) at both six (P = 0.039) and eighteen months (P = 0.039), with mean amplitudes suggesting offspring of mothers with gestational diabetes exhibit greater neuronal activity to standard stimuli and less to oddball stimuli. Associations between 2-hour maternal glucose levels and the difference in EPmax amplitude were marginal at 6 months [adjusted β = -0.19 (95% CI: -0.42 to +0.04) μV, P = 0.100] and significant at 18 months [adjusted β = -0.27 (95% CI: -0.49 to -0.06) μV, P = 0.014], and the EPmax amplitude difference (oddball-standard) associated with the Bayley Scales of Infant and toddler Development-III cognitive score at 24 months [β = 0.598 (95% CI: 0.158 to 1.038), P = 0.008].

**Conclusion:**

Gestational diabetes and maternal blood glucose levels are associated with offspring neuronal activity during an attentional task at both six and eighteen months. Such electrophysiological differences are likely functionally important, having been previously linked to attention problems later in life.

## Introduction

The incidence of diabetic pregnancies is increasing [1, 2], with research reporting adverse effects on offspring perinatal [3] and long-term health [4], including poorer cognitive outcomes [5–8]. However, confounders such as socioeconomic status may make some findings difficult to interpret [9, 10]. Since these confounders accumulate with age, the effects of diabetic pregnancies are better investigated during early childhood. To date, at least eight studies have focused on children aged two or younger, and most observed an association between diabetic pregnancies [gestational diabetes mellitus (GDM) or pre-gestational diabetes] and poor cognitive performance [7, 8, 11–16]. However, the majority of such research, including *all* research in infants (under 12 months), has focused on memory [8, 11–15] and all but one infant study, used event related potentials (ERPs).

ERPs reflect the coordinate neuronal activity that occurs in response to experimental stimuli. They reflect processing as it unfolds, and an ERP that occurs rapidly after stimulus onset is likely to reflect sensory processes, while slightly later occurring ERPs may reflect attention, and later components are often considered indicative of memory [17]. Several ERP memory studies report an influence of diabetic pregnancies not only on late occurring ERPs, but also earlier ERPs, believed to reflect attentional processes [11–13]. These findings are consistent with research in children, where the impact of diabetic pregnancies has been found to extend beyond memory, including language [7], motor skills [18] and general cognition [5, 13]. However, findings with behavioral measures have been inconsistent. While some groups observed poorer scores on the Mental Development Index of the Bayley Scales of Infant Development (BSID) [12, 13], others report no significant differences [11, 19]. Aside from the possibility of differential cross-study exposure to accumulating confounding factors, one potential reason for inconsistencies is that in early life, the detection of effects from maternal hyperglycemia may require electrophysiological testing methods. It has been previously suggested that ERPs may detect cognitive differences in infants of GDM mothers better than other tests [11, 12]. Seven previous ERP studies have reported associations between diabetic pregnancy and aspects of neurophysiology reflecting memory updating [11–16, 20]. Four also included behavioral measures of infant memory, such as deferred imitation, but only two of these observed significant associations with GDM [13, 20]. In one of the studies reporting no effects of GDM on behavioral (BSID) performance, GDM associated changes in electrophysiology were themselves predictive of BSID scores [15].

In our study, we utilized behavioral and eye tracking indicators of infant memory and attention as well as an electrophysiological attentional task, to address three important gaps in the current literature. First, we examined whether GDM influences attention, as measured by ERP during the auditory oddball task. Second, to assess a possible dose-response effect, we investigated whether maternal blood glucose levels predict infant cognitive functioning, across the normal and GDM ranges. Third, as past research has shown inconsistent results on GDM and behavioral measures of cognition, we explored whether GDM associates with such measures, and in keeping with de Regnier et al. [15], we aimed to determine whether any observed GDM related differences in ERPs were predictive of BSID cognitive score.

## Materials and Methods

### Participants

Pregnant women in their first trimester were recruited at Singapore’s Kandang Kerbau Women's and Children's Hospital and National University Hospital between June 2009 and September 2010 (n = 1247) to join the Growing Up in Singapore Towards healthy Outcomes (GUSTO) prospective birth cohort study. Women with type 1 diabetes mellitus, on chemotherapy or psychotic medications were not eligible to participate. [21] Infants were delivered between November 2009 and May 2011. A subgroup of mother and child dyads took part in neurocognitive assessments between June 2010 and May 2013 when the children were 6 months (n = 473), 18 months (n = 431), and 24 months (n = 514) of age [22].

Participants with known type 2 diabetes and/or pregnancy complications (e.g., preeclampsia) other than gestational diabetes, multiple pregnancy (i.e twins), offspring who received a last recorded Apgar score of less than 9, had a birthweight less than 2500 g or gestational age of less than 37 weeks, were conceived by *in vitro* fertilization, and/or were tested outside the window periods (6 month visit: 6 months ± 2 weeks; 18 month visit: 17 to 19 months and 24 month visit: 23 to 25 months) were excluded. 473 subjects who underwent one or more of the neurocognitive assessments met the eligibility criteria: 357 at 6 months, 327 at 18 months and 398 at 24 months. Non-participation at each stage was detailed in Cai et al. [22], briefly non-participation could be due to lack of interest, busy schedules, inability to reach the participants or their dropout from the cohort study.

The study was approved by the National Healthcare Group Domain Specific Review Board (reference number D/09/021) and the Sing Health Centralized Institutional Review Board (reference number 2009/280/D). All participants gave informed written consent prior to their participation.

### Diagnosis of GDM and Blood Glucose Measurement

GDM was diagnosed at 26–28 weeks gestation using a 75-g oral glucose tolerance test after overnight fasting. Blood glucose levels were collected twice (fasting and 2-hour post-glucose) to minimize subject burden. Therefore, we used the 1999 World Health Organization (WHO) diagnostic criteria, which defines GDM as ≥7.0 mmol/L for fasting glucose and/or ≥7.8 mmol/L for 2-hour post-glucose [23, 24]. Women with GDM were subsequently managed according to standard hospital protocols.

### Cognitive Outcome Measurements

Neurocognitive assessments were carried out at 6, 18 and 24 months. Details of the testing procedures have been previously described [22]. Briefly, we assessed memory (habituation at 6 months; deferred imitation at 6, 18 and 24 months; relational binding at 6 months) and attention [visual expectation and auditory oddball ERP at 6 and 18 months], together with a global measure based on the Bayley Scale of Infant and Toddler Development, Third Edition (BSID-III) which includes five subscale scores for cognition, expressive and receptive language and both fine and gross motor function [25].

### Auditory Oddball (Event-Related Potentials)

The detailed protocol was previously described [22] (details in S1 Supporting Information). Briefly, the children were presented with the sound syllables “ma” and “na.” The presentation of “ma” vs “na” as the standard sound was counterbalanced. Stimuli (475ms each) were presented in 4 blocks (total of 1600 trials, with a 800ms inter-stimulus interval and the oddball sound was played for 15% of the trials). Data were collected with a NetStation 300 (Electrical Geodesics, Inc., Eugene, OR) within a range of 0–100 Hz, initially referenced to the vertex, via a 128 channel system.

Two distinct ERP components were observed in the whole GUSTO sample, an early negative (EN) deflection (6 months: 8 to 228ms; 18 months: 8 to 218ms) followed by an early positive (EP) peak (6 months: 128 to 508ms; 18 months: 98 to 438ms). The most negative and positive points within the relevant time windows were extracted for EN and EP respectively for all participants.

### Other Data

Antenatally, mothers completed questionnaires regarding demographic and socioeconomic status, medical histories, smoking and alcohol exposures as well as maternal mood and anxiety [Edinburgh Postnatal Depression Scale (EPDS) [26] and the State-Trait Anxiety Inventory (STAI) [27] respectively]. Gestational weight gain z scores were derived as described previously (28). Midwives recorded birth outcomes (e.g., birthweight and gestational age) and Apgar scores at delivery.

### Statistical Analysis

Continuous and categorical infant and maternal characteristics were compared between GDM and non-GDM mothers using independent sample t-test and chi-square tests, respectively. Multivariable linear regression models were used to assess the effect of glucose levels (continuous variable) or GDM status (categorical variable) on each behavioral outcome and unstandardized coefficients are reported. These models were adjusted for ethnicity, gestational age and sex of the offspring, as well as the following maternal covariates: maternal education, age, pre-pregnancy body mass index (BMI), gestational weight gain z-score and imputed (imputation by hot deck imputation for missing items) antenatal anxiety (STAI) scores. Procedural variables (e.g. infant sleep state at test, stimuli type) associated with predictor and outcome variables were also included. Maternal age, education, ethnicity, gestational age, pre-pregnancy BMI and gestational weight gain were selected as they are known to affect both GDM risk and offspring cognition independently of GDM. We included maternal antenatal anxiety as a covariate, because psychological stress may influence glucose tolerance [28] and evidence suggests that maternal mood during pregnancy can influence offspring cognition [29]. The ratio of subjects to the number of independent variables (covariates and exposure of interest) for all the final models ranged from 10.2 to 35.5. Models were re-run using bootstrapping for all significant and marginal (P<0.10) findings, to rule out false positive findings associated with any deviance from model assumptions. Bootstrapping yielded similar results (available upon request) and where the results changed from P<0.10 to P≥0.10, the results are reported. For analysis of ERP data at 6 and 18 months, we performed repeated-measures analysis of variance (ANCOVA) including the aforementioned covariates. Separate models considered component (i.e., EN and EP) amplitudes and latencies. Given our primary interest in attention (i.e., reflected here as neuronal activity to different stimuli), within subject ERP predictors were specified as stimuli (oddball vs standard), stimuli*electrode region (frontal vs central), stimuli*hemisphere (left vs right) and stimuli*hemisphere*region. Likewise, only covariate interactions including stimuli were retained in the models. Interaction terms associated (p<0.10) with GDM were further examined with the same repeated measures ANCOVA approach, with further stratification of variable(s). Differences (p<0.10) between GDM and controls were followed up with multivariable linear regression analyses to examine the magnitude of difference in stimuli responsiveness according to GDM status. Repeated measure ANCOVA was used to examine the within group comparison of response to oddball and standard stimuli, followed by pairwise comparison between stimuli. In cases where EP effects (p<0.10) were observed, sensitivity analyses were conducted using the difference between the EN trough and EP peak (EN-EP complex). In addition, post hoc multivariable linear regression analyses were conducted to examine the prediction from blood glucose levels to differential neuronal activity (oddball-standard).

Multivariable linear regression models were used to assess the association between ERP variables that were associated with GDM status and BSID-III cognitive score, with the same covariates adjusted above.

Data were missing on maternal age in 1.7% (n = 8), antenatal EPDS in 3.2% (n = 15), STAI scores in 7.0% (n = 33), household income in 7.6% (n = 36) and maternal education in 3.0% (n = 14) of cases. For all significant and marginal (P<0.10) findings, models were re-run with multiple imputation. Multiple imputation of missing data (maternal education, maternal antenatal anxiety scores) using chained equations imputation (20 imputations) yielded similar findings to those in subjects with complete data (imputed data available upon request). All analyses were done with SPSS version 22.0 (IBM, Armonk, NY, USA).

## Results

### Participant Characteristics

Mother-child pairs who participated in the neurocognitive assessments were comparable to the non-participants in ethnicity, household income, maternal age and education [22]. Mothers who participated in the neurocognitive assessments displayed more anxiety and depression traits during pregnancy than non-participants [22]. Non-participation at each stage was previously described [22].

Seventy four and 399 offspring born to mothers with and without GDM respectively were included in this analysis. Maternal age (33.6 ± 4.8 vs 30.0 ± 5.1 years; P<0.001) and education level were higher (P = 0.021) in mothers with GDM (Table 1). A lower proportion of male infants were born to mothers with GDM (41.9% vs 56.1%; P = 0.024). GDM and control groups were comparable on other variables (Table 1).

**Table 1:** Comparison of baseline characteristics of participants with and without GDM.

		oGDM (N = 74)	Control (N = 399)	P value
**Maternal Variables**				
**Age (years)**		33.6 ± 4.8	30.0 ± 5.1	<0.001
**Pre-Pregnancy BMI (kg/m**^**2**^**)**		23.6 ± 4.4	22.8 ± 4.6	0.148
**Antenatal Maternal EPDS score**		7.4 ± 4.3	7.8 ± 4.6	0.424
**Antenatal Maternal STAI-state score**		34.1 ± 9.6	35.0 ± 9.7	0.458
**Antenatal Maternal STAI-trait score**		35.3 ± 8.8	37.2 ± 9.0	0.100
**Fasting glucose (mmol/L)**		4.60 ± 0.61	4.32 ± 0.42	<0.001
**120min glucose (mmol/L)**		8.71 ± 1.11	5.91 ± 0.99	<0.001
**Gestational weight gain z-score**		-0.84 ± 1.06	-0.86 ± 1.05	0.847
**Alcohol consumption during pregnancy, n (%)**		1 (1.4)	9 (2.3)	0.619
**Smoked during pregnancy, n (%)**		1 (1.4)	11 (2.8)	0.778
**Infant Variables at birth**				
**Gestational Age (weeks)**		38.8 ± 1.0	39.0 ± 1.0	0.204
**Birth Weight (g)**		3195 ± 376	3178 ± 366	0.702
**Birth Weight >4000g, n (%)**		1 (1.4)	9 (2.3)	0.619
**Birth Length (cm)**		49.2 ± 2.0	48.8 ± 2.0	0.100
**Sex of child (Male), n (%)**		31 (41.9)	224 (56.1)	0.024
**Ethnicity, n (%)**				0.179
	Chinese	46 (62.2)	211 (52.9)	
	Malay	15 (20.3)	123 (30.8)	
	Indian	13 (17.6)	65 (16.3)	
**Maternal Education, n (%)**				0.021
	Primary	3 (4.1)	18 (4.5)	
	Secondary	9 (12.2)	99 (24.8)	
	Diploma/ Technical Education	26 (35.1)	145 (36.3)	
	University	32 (43.2)	120 (30.1)	
	Postgraduate	3 (4.1)	4 (1.0)	
	Missing Data	1 (1.4)	13 (3.3)	
**Household Income, n (%)**				
	$0–999	0 (0.0)	14 (3.5)	0.425
	$1000–1999	8 (10.8)	47 (11.8)	
	$2000–3999	19 (25.7)	116 (29.1)	
	$4000–5999	19 (25.7)	86 (21.6)	
	>$6000	23 (31.1)	105 (26.3)	
	Don’t know/ Refused to answer/Missing data	5 (6.8)	31 (7.8)	

Data presented as mean ± standard deviation. oGDM- offspring of mothers with Gestational Diabetes Mellitus, EPDS-Edinburgh Postnatal Depression Scale, STAI- State-Trait Anxiety Inventory

### Event-Related Potential (ERP) Assessment of Attention

**Fig 1:**
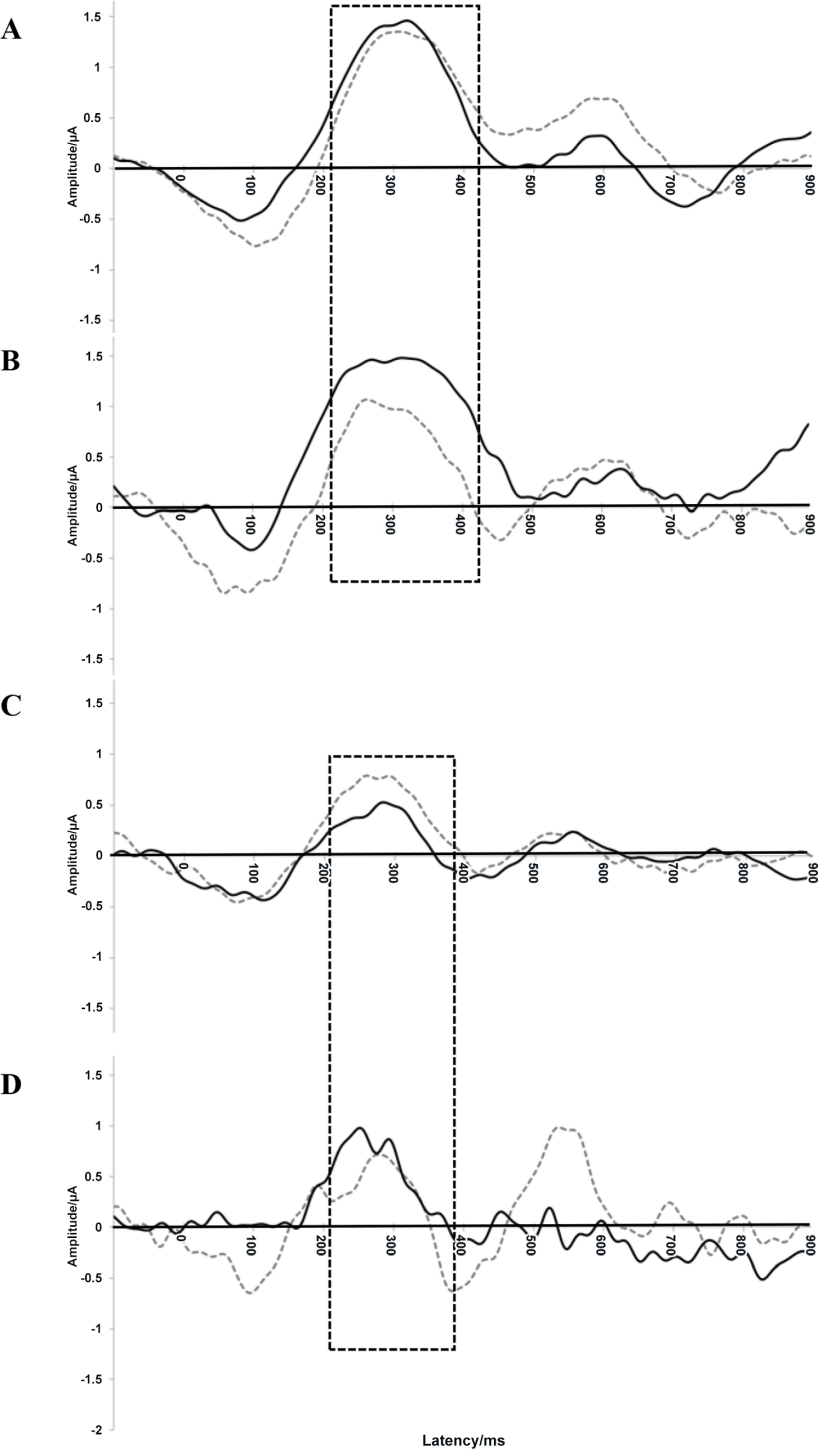
**Composite (grand average) ERP recording of electrodes in the left hemisphere of 6-month-old controls (A) and offspring of mothers with GDM (oGDMs) (B), as well as 18-month-old controls (C) and oGDMs (D).** The solid line represents the standard stimulus and the dotted line represents the oddball stimulus. The boxed region of the graph corresponds to the EPmax, where the degree of differential neuronal activity (oddball-standard) significantly varied according to GDM status.

Fig 1 illustrates the grand averaged ERP recording of electrodes from the left hemisphere, to standard and oddball stimuli at 6 and 18 months.

No significant main or interaction effects of GDM were observed at 6 and 18 months with regards to the EN component amplitude or latency, nor with the EP latency (data not shown). However, we observed a stimuli*hemisphere*GDM group interaction (ANCOVA P = 0.080) for the early positive (EPmax) amplitude in 6-month-olds. The stimuli*GDM interaction was significant over the left hemisphere (ANCOVA P = 0.039) but not the right (ANCOVA P = 0.964). Indeed, GDM associated with differential processing of oddball and standard stimuli (see Table 2 and S2 Supporting Information). In the left hemisphere, the activity to standard stimuli was greater, though non-significantly so, amongst offspring of mothers with GDM (oGDM) (mean ± SD: 3.23 ± 0.30 μV) compared to controls (2.95 ± 0.17 μV) (P = 0.398). Neuronal activity to oddball stimuli was lesser, albeit non-significantly so, amongst oGDMs (2.62 ± 0.30 μV) than their control counterparts (3.10 ± 0.17 μV) (P = 0.153).

**Table 2:** Effect of GDM on difference in EPmax amplitude towards oddball and standard stimuli, stratified by hemispheres.

	Unadjusted difference in the mean amplitudes to oddball and standard stimuli ^a^		Adjusted difference in the mean amplitudes to oddball and standard stimuli ^a^ ^b^	
	Control	oGDM	Control	oGDM
**6 months (n = 104 control, 25 GDM)**				
Left Hemisphere (μV)	0.06 (1.51)	-0.60 (1.47)*	0.15 (1.58)	-0.62 (1.51)*
Right Hemisphere (μV)	0.06 (1.53)	-0.21 (1.56)	-0.16 (1.66)	-0.18 (1.65)
**18 months (n = 87 control, 15 GDM)**				
Left Hemisphere (μV)	0.23 (1.44)	-0.32 (1.25)	0.27 (1.72)	-0.66 (1.44)*
Right Hemisphere (μV)	0.16 (1.47)	-0.04 (1.57)	0.09 (1.96)	-0.20 (1.69)

^a^ Stimuli difference = oddball—standard

*P<0.05 compared to control. Data presented as mean (SD)

^b^ Adjusted for maternal age, maternal education, sex and gestational age of child, ethnic group, 26 weeks STAI-state, maternal pre-pregnancy BMI and gestational weight gain at 26 weeks gestation.

Initial analyses of the 18 month data did not reveal any main or interaction effects of GDM on the EN or EP. Nevertheless, given the six month findings, we examined whether, a similar effect of stimuli*GDM in the left hemisphere would be observed. Indeed, the stimuli*GDM interaction remained significant over the left hemisphere (ANCOVA P = 0.039) and not the right (ANCOVA P = 0.576). Compared to controls (mean ± SD: 1.85 ± 0.13 μV), oGDMs (2.44 ± 0.26 μV) showed significantly more neuronal activity towards standard stimuli (P = 0.038). Though not significant, oGDMs also showed lesser neuronal activity towards oddball stimuli (1.78 ± 0.35 μV) compared to controls (2.12 ± 0.18 μV) (P = 0.376).

**Fig 2:**
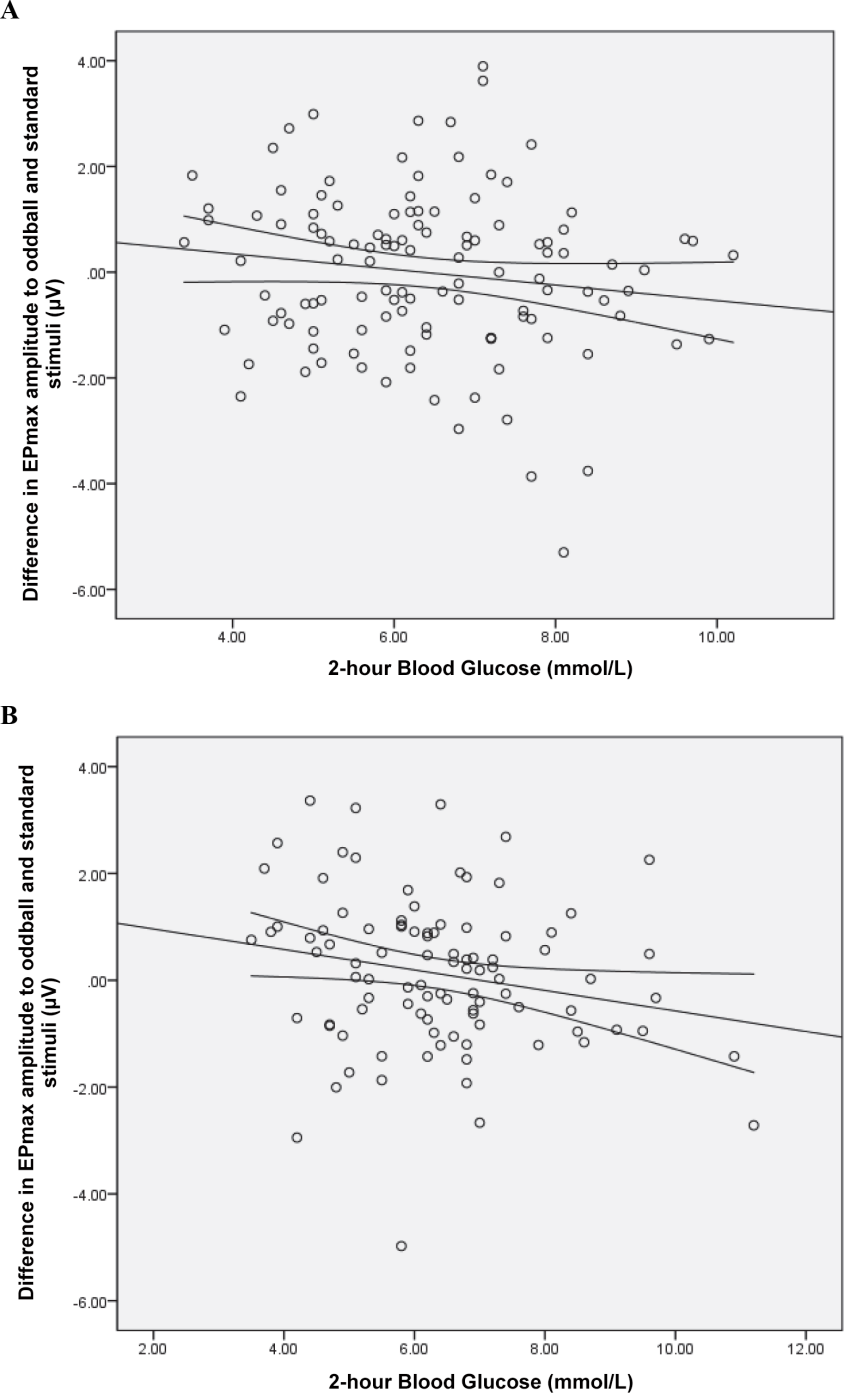
Scatterplots of stimuli difference (oddball-standard) in EPmax amplitudes, over the left hemisphere. Stimuli difference in EPmax amplitudes over the left hemisphere in A) 6 months and B) 18 months old infants against maternal 2-hour plasma glucose at 26–28 weeks gestation. Curves lines correspond to 95% confidence interval of the mean.

A dose-response relationship was observed between the 2-hour blood glucose concentration and the difference between the EPmax to oddball and standard in the left hemisphere (Fig 2). This relationship showed a marginal trend at 6 months [adjusted β = -0.19 (95% CI: -0.42 to 0.04) μV, R^2^ = 0.214] (Fig 2A) and was significant at 18 months [adjusted β = -0.27 (95% CI: -0.49 to -0.06) μV, R^2^ = 0.182] (Fig 2B). An increase in maternal 2-hour blood glucose level (per mmol/L) was associated with a non-significant decrease in neuronal activity towards the oddball stimulus at both time points [6-month adjusted β = -0.046 (95% CI: -0.254 to 0.163) μV; 18-month adjusted β = -0.131 (95% CI: -0.318 to 0.056) μV], and a non-significant increase in neuronal activity towards the standard stimulus at six months [6-month adjusted β = 0.145 (95% CI: -0.062 to 0.351) μV) as well as a significant increase in neuronal activity towards the standard stimulus at 18-month adjusted β = 0.141 (95% CI: 0.003 to 0.280)μV]. Bootstrapped results show that at 18 months, the increase in neuronal activity towards the standard stimulus was no longer statistically significant (P = 0.150).

Sensitivity analysis using the EN-EP complex are reported in the S2 Supporting Information. The associations between EPmax amplitude and GDM status (or maternal blood glucose concentration) did not differ significantly by sex of the child or ethnicity.

### Behavioral Measures

No significant effects of GDM or maternal blood glucose levels were observed on any behavioral measures (see S2 Supporting Information) except that oGDMs (547 ± 29ms) responded in a marginally shorter time to the stimuli compared to controls (606 ±16ms) (P = 0.061) during Visual Expectation pattern trials at 18 months. There was a trend association between BSID-III fine motor scaled score and GDM, where the oGDM has a marginally higher fine motor score (11.50 ± 0.35) compared to controls (10.88 ± 0.17) (P = 0.097) but with bootstrapping, the association was lost (P = 0.10).

However, we noted that the EPmax amplitude difference over the left hemisphere (oddball-standard) in 6 months old infants was significantly associated with the Bayley cognitive score (β = 0.598, P = 0.008) (i.e a more positive EPmax amplitude difference, as observed in controls, is associated with a higher cognitive score). A similar but non-significant trend was also observed at 18 months (β = 0.228, P = 0.319).

## Discussion

We found that GDM is associated with alterations in neurophysiology previously reported as relevant to attention [30] and distractibility [31]. We also found that these neurophysiological differences, observed at 6 and 18 months of age, were associated with maternal 2-hour blood glucose concentrations at 26–28 weeks of pregnancy. These results are consistent with a recent report wherein GDM impairs human fetal brain activity, with slower postprandial auditory evoked responses [32]. In keeping with some [11, 12] but not all [8, 13] previous studies, we did not detect much influence of GDM status or maternal blood glucose on behavioral tests of cognition in the first two years of life. GDM status was not predictive of attention, memory-related behaviors or overall development assessed by BSID-III except faster reaction time towards the stimuli during the visual expectation task, which indicate faster attentional orienting [33]. Similarly, when taking part in behaviorally based (not eye tracking) forms of attentional tasks, children with attention deficit hyperactivity disorder (ADHD) have sometimes been reported to respond more quickly, compared to controls [34]. Still, quicker processing speed during visual expectation has also been suggested to be an important component of better infant cognition [35]. Thus, the meaning of the observed association between GDM and visual expectation reaction speed is unclear.

Overall, however, similar to findings by Nelson et al., [11, 12] our results may suggest the sensitivity of electrophysiological measurement tools to detect effects of GDM. Our findings extend past findings by suggesting an impact of maternal glycemia on attentional processing, even at early stages of development.

In particular, during our ERP tasks at both 6 and 18 months, oGDMs differed from their control counterparts in the way they processed oddball versus standard sounds, especially over the left hemisphere—the hemisphere generally considered to be responsible for attention and processing of speech sounds [36] used as stimuli in our ERP tasks. To our knowledge, no previous research has assessed the influence of GDM on infant attention in the first two years of life, despite many reports of an increased prevalence of attention problems amongst offspring of mothers with diabetic pregnancies [6, 18, 37–39]. Prior research in infants has focused largely on memory, likely influenced by knowledge of the pathophysiology of diabetic pregnancy (fetal hyperglycemia, hypoxemia and iron deficiency and neonatal hypoglycemia) [40]. Animal research indicates that brain regions like the hippocampus and striatum, which are important for memory processing, are particularly sensitive to prenatal iron deficiency [41, 42]. Nevertheless, our observations with the ERP may help to bridge the evidence gap between memory effects reported in infancy [8, 11–15] and later childhood and adult studies focusing on attention.

ERP studies utilizing memory paradigms have observed differences in early ERP components thought to reflect differences in attention during a memory task [11–13]. Past research used paradigms that compare neural responses to a stimulus that is well-encoded and familiar (voice or face of the mother) to response to a novel stimulus (voice or face of a stranger). Thus, differences in attention allocation are presumed to be reflective of differences in the strength with which the familiar stimulus was encoded into memory. As familiarization and encoding occur any time prior to testing, it is not possible to specifically examine the concurrent influence of GDM on attentional and/or memory processes at the time of testing.

In our testing paradigm, instead of using pre-familiarized versus novel stimuli, we presented two familiar phonemes at different rates. Thus the standard phoneme, which was presented 85% of the time, should become familiar over the course of the testing. Attentional processing is expected to eventually decline, a pattern consistent with expectations concerning neural habituation, defined as a decrease in neural response resulting from repeated stimulation [43]. Here, oGDMs responded to the familiar stimuli to a relatively greater extent than controls—suggesting failure to encode the repeated sound, and correspondingly, persistence in attentional processing manifest during the attention-relevant task. Greater EPmax amplitude to the standard stimuli may imply poorer habituation in oGDMs, possibly indicating weaker adaptive brain functioning [44], as well as memory [45]. This lends support to the well-reported effect of diabetic pregnancy on infant memory [8, 11–15]. Future studies should assess whether GDM-related early memory deficits underlie subsequent difficulties in attentional processing.

Our results suggest the importance of electrophysiological methods for observing early-life effects of GDM. While some investigators have observed differences in behavioral tasks involving memory [8, 13, 20], we did not detect significant differences in any of our behavioral memory tasks. Other studies have reported differences in oGDMs, based on ERP, but not on behavioral measures [11, 12], suggesting that behavioral measures may be less sensitive than ERP for detecting subtle GDM effects. Thus, as suggested by Nelson and colleagues [11, 12], our findings confirm that electrophysiology may be a better tool for detecting subtle GDM effects. The women in our cohort were universally screened for GDM and followed up with standard management and these may have contributed to the absence of associations with behavioral outcomes. Although we have no data on compliance with GDM treatment, the comparable birthweights and rate of macrosomia (birthweight of >4000g) (Table 1) of oGDMs and controls suggest that the mothers in our cohort had well-controlled GDM. Several studies that reported significant effects of GDM on behavioral outcomes also observed higher birthweights in the offspring [5, 8, 20].

Our findings suggest that even well-controlled GDM can result in subtle differences in the offspring’s neurodevelopment. These subtle differences may be important, as electrophysiological response during the oddball task has been associated with later adverse clinical outcomes, including ADHD [30, 46] which has been previously reported to be more prevalent in offspring of mothers with diabetic pregnancies [6, 18, 37–39]. Likewise, as observed here, differential ERPs during the oddball task may predict performance on a developmental screening tool like BSID-III [15, 47].

Our study has several strengths, including the use of a variety of measures to test specific cognitive processes in the first two years of life, while most studies focused on memory and general cognition. Our study is based on a large Asian cohort, which is important as Asians are at higher risk of GDM than their Caucasians counterparts [48]. Our study controlled for many potential confounders and still observed associations between GDM and offspring neurocognitive outcomes. Finally, ours is one of the few studies to demonstrate a dose-dependent association of maternal blood glucose with neurocognitive outcomes in the offspring, which suggests a beneficial impact of good glycemic control even below the diagnostic threshold for GDM.

We analyzed many outcomes and found significant GDM related differences only in 2 of 14 cognitive tasks. While we cannot rule out the possibility of chance findings, it is important to note that the associations we observed with the ERP task are consistent with past research [11–13]. Moreover, we observed consistent differences in ERP results at both 6 and 18 months of age as well as a dose-response relation with maternal 2-h post OGTT blood glucose, thus the findings are unlikely to have occurred by chance. It is of note that amongst controls, we only observed significantly greater neuronal activity to the oddball as compared to the standard stimuli at six, and not eighteen, months. However, individual differences in passive auditory tasks are sometimes observed despite a lack of statistically significant differences in neuronal activity by stimuli type [49]. Moreover, amplitudes of positive auditory oddball components have been found to decrease after nine months of age [50] and an eventual lack of differentiation between oddball and standard sounds may indicate a developmental shift from the detection of any (acoustic) deviance to the more specific detection of context relevant change [51]. Although women whose children participated in the neurocognitive assessments differed in some respects from non-participants [22], it is unlikely that these differences would bias our findings. As we recruited the women in their first trimester, we were unable to test and rule out undetected pre-gestational diabetes within the GDM group. Another limitation is the lack of strong indicators of glycemic control (eg HbA1c) to be able to definitively suggest if the GDM cases in our study were well-controlled.

In conclusion, we observed an association between GDM and attention in offspring under 2 years of age. ERP measures may be sufficiently sensitive to detect subtle differences in oGDMs during early life, particularly in well controlled GDM. If the association we observed between GDM and altered offspring attention persists at later stages of development, pre-conception and early pregnancy prevention programs should be considered for women at risk for gestational diabetes, as should interventions for their offspring.

## Supporting Information

S1 Fig Flow chart of subject participation. (PDF)

S1 Supporting Information Supplementary methods. Detailed protocol for auditory oddball (event related potentials).
(DOCX)

S2 Supporting Information Supplementary results. (DOCX)
