# Supplementary figures and images for: The Influence of Gestational Diabetes on Neurodevelopment of Children in the First Two Years of Life: A Prospective Study
Source: PLoS One. 2016 Sep 7;11(9):e0162113. doi: 10.1371/journal.pone.0162113 (PMC5014336; doi:10.1371/journal.pone.0162113)

S1 Fig. Flow chart of subject participation.

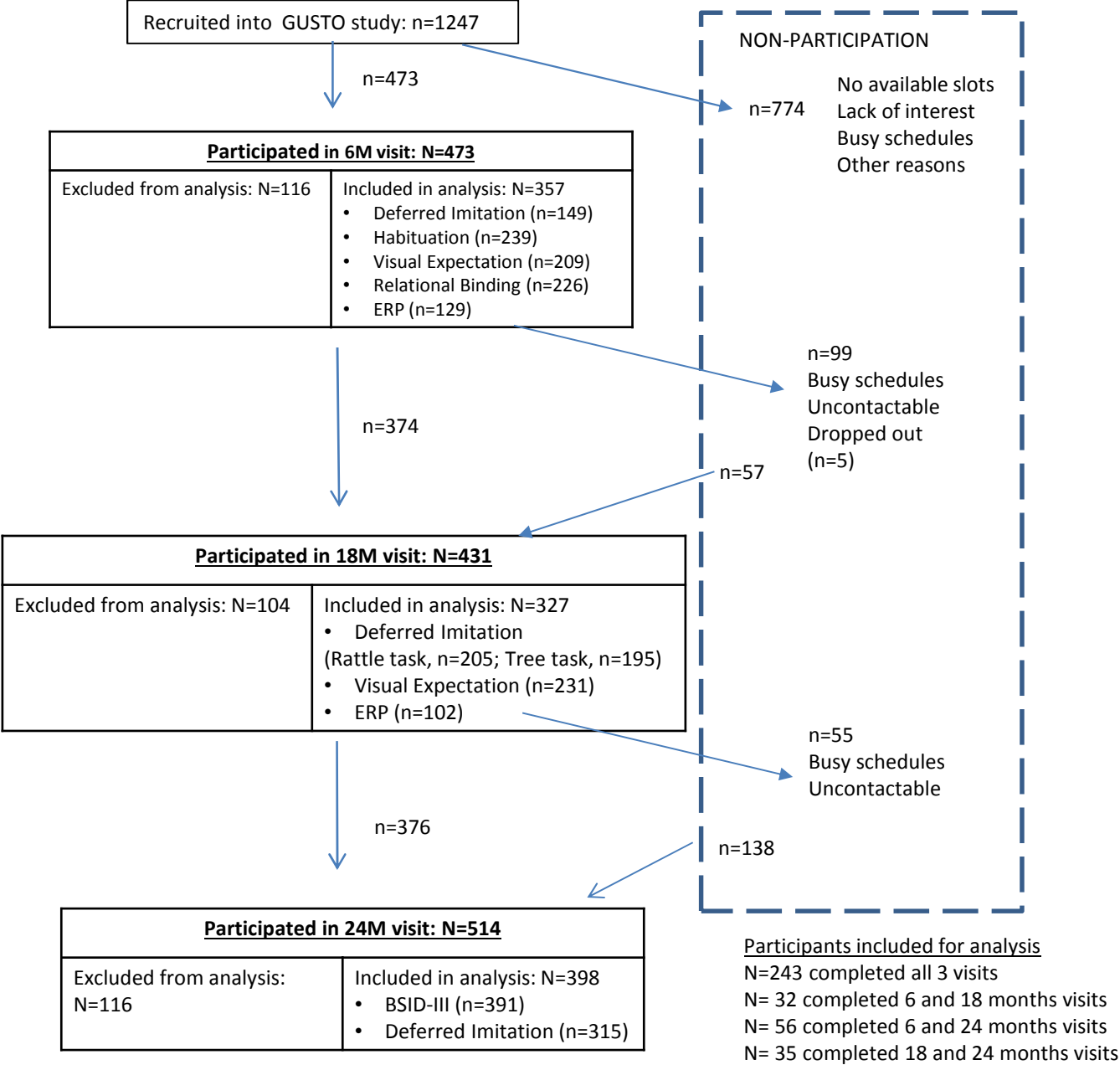

Supplement: S1 Fig — (PDF) [file pone.0162113.s001.pdf]
