# Supplementary material for: The Influence of Gestational Diabetes on Neurodevelopment of Children in the First Two Years of Life: A Prospective Study
Source: PLoS One. 2016 Sep 7;11(9):e0162113. doi: 10.1371/journal.pone.0162113 (PMC5014336; doi:10.1371/journal.pone.0162113)
Supplement: S1 Supporting Information — Detailed protocol for auditory oddball (event related potentials). (DOCX) [file pone.0162113.s002.docx]

**S1 Supporting Information. Supplementary methods.**

***Auditory Oddball (Event-Related Potentials)***

The detailed protocol was previously described (22). In brief, the children were presented with a stream of sound syllables “ma” and “na.” The presentation of “ma” vs “na” as the standard sound was counterbalanced. Stimuli (475ms each) were presented in 4 blocks (total of 1600 trials, with a 800ms inter-stimulus interval, an in which the oddball sound was played for 15% of the trials). EEG’s were collected within a range of 0-100 Hz, initially referenced to the vertex, via a 128 channel system. Post-collection, a 1-30 Hz band filter was applied. All oddball trials, and 240 randomly selected standard trials were segmented into 1000ms segments with a 8ms offset, baseline corrected, and run through an automated (Net Station, Electrical Geodesics, Inc., Eugene, OR) artefact detection script. Next, we hand-edited files to confirm or reject the automated assessment of eye movements, eye blinks, and/or high frequency wave or non-brain wave noise. Then, trials with greater than 18-20 of bad channels were omitted from calculations. In retained files, channels deemed corrupt were replaced with interpolated results from surrounding electrodes. Data were then averaged, per individual, within each experimental condition (oddball or standard), re-referenced to the average of 124 electrodes and once again baseline corrected. Finally, each subject’s resultant averaged file was then double checked for artefacts and excessive noise. Only individual averages free of excessive artefacts or noise were included in analyses. Data was extracted from 44 channels in the frontal and central regions.

Individual averages were additionally averaged to create a “grand average” important to identifying “peaks” and “troughs” in the averaged time-locked wave. Within the grand average, two distinct ERP components were observed, an early negative (EN) deflection followed by an early positive (EP) peak. Visual inspection of individual averages suggested that the negative deflection occurred between 8 to 228 ms and 8 to 218 ms at 6 and 18 months respectively. EP occurred between 128 to 508 ms and 98 to 438 ms at 6 and 18 months respectively. In recognition of the large individual variation in the timing of these components, we then extracted the most negative point within the relevant time window (e.g.8 to 228 ms at 6 months) as a measure of the early negativity and the most positive point within the relevant time window (e.g. 98 to 438 ms for the EP at 18 months) as a measure of the early positivity. Following data processing, EGI released an announcement (EGI Advisory Notice, 28 August 2014), affecting almost all researchers using their 300 or 400 series equipment and software, indicating an additional, and previously unidentified, delay in the time axis, here amounting to 8ms. This delay was uniform across participants and should not influence relative differences according to subject group, stimulus type, etc, and is likely of most interest only to electrophysiologists interested in the absolute millisecond timing of observed components. Above, to be consistent with ours and others past research published prior to the recognition of the delay, we reported the extracted value and time windows, not accounting for this delay.
