# Supplementary material for: The Influence of Gestational Diabetes on Neurodevelopment of Children in the First Two Years of Life: A Prospective Study
Source: PLoS One. 2016 Sep 7;11(9):e0162113. doi: 10.1371/journal.pone.0162113 (PMC5014336; doi:10.1371/journal.pone.0162113)
Supplement: S2 Supporting Information — (DOCX) [file pone.0162113.s003.docx]

**S2 Supporting Information. Supplementary results.**

***Event-Related Potential (ERP) Assessment of Attention***

Within Group Stimuli Effects. At six-months, repeated measures ANCOVA revealed an overall effect of stimuli in controls (P=0.002) but not in oGDMs (P=0.969). Posthoc pairwise comparisons, excluding ANCOVA interactions between stimuli type and covariates, showed that the EPmax amplitude difference over the left hemisphere is positive in control (Fig 1A, oddball>standard, P=0.368) and negative in oGDM groups (Fig 1B, standard>oddball, P=0.187). At 18months, no significant overall effects of stimuli were observed in either controls or oGDMs. As depicted in Figure 1D, 18 months oGDMs displayed a greater amount of firing to the standard as opposed to oddball stimuli (standard>oddball, P=0.715) - whereas control offspring continued to show greater neuronal firing towards oddball stimuli (oddball>standard, P=0.316) (Fig 1C).

Repetition of Main Analyses, utilizing EN-EP as the outcome variable. As discussed, to ensure the specificity of our findings, the analyses reported in the main text were repeated using the EN-EP (rather than the EP) as the outcome and revealed a stimuli x GDM interaction (P=0.065) in six months old infants. Among controls, there was a significant effect of stimuli on EN-EP complex amplitude (P=0.002), with greater amplitude for oddball compared to standard, but not in oGDMs. At 18 months, there were neither marginal (P<0.10) nor significant main effects of GDM or interaction effects with GDM. There was no significant effect of condition on EN-EP complex amplitude in controls. EN-EP complex amplitude difference (oddball-standard) did not associate significantly with fasting or 2h blood glucose at both six and 18 months, but the trend with 2h blood glucose is similar [6 months: β= -0.34 (95% CI: -1.05 to 0.37) µV; 18 months: β= -0.40 (95% CI: -0.98 to 0.18) µV] to what was observed with EPmax.

***Attention (Looking behavior)***

At both 6 and 18 months of age, no differences were observed for the visual expectation task. Controls and oGDMs did not differ significantly in their proportion look at correct location when the stimuli appeared on the screen or during anticipatory period. In terms of reaction time to the stimuli, there was no difference between both groups at 6 months (P=0.794) but at 18 months oGDMs responded in a shorter time to the stimuli compared to controls (Control: 606 ±16ms vs GDM: 547 ± 29ms, P=0.061) during pattern trials. No dose-response effect was observed between any of the visual expectation variables and maternal blood glucose concentrations.

***Memory Tasks (Looking and Motor Behaviors)***

GDM and maternal blood glucose concentrations were not significantly associated with any of the memory tests (i.e., habituation, relational binding and deferred imitation) at any time point (data not shown).

***Bayley Scales of Infant and Toddler Development***

The two groups scored similarly in the cognitive, language (expressive and receptive) and motor (fine and gross) skills domains (data available on request). Maternal blood glucose concentrations were not significantly associated with any of the domains in BSID-III.
